# Characteristics of Ocular Findings of Patients With Coronavirus Disease 2019 (COVID-19) in Hubei Province, China

**DOI:** 10.1001/jamaophthalmol.2020.1291

**Published:** 2020-03-31

**Authors:** Ping Wu, Fang Duan, Chunhua Luo, Qiang Liu, Xingguang Qu, Liang Liang, Kaili Wu

**Affiliations:** 1 Department of Ophthalmology, The First College of Clinical Medical Science, Yichang Central People’s Hospital, China Three Gorges University, Yichang, China; 2 Zhongshan Ophthalmic Center, State Key Laboratory of Ophthalmology, Sun Yat-Sen University, Guangzhou, China

## Abstract

**Importance:**

While the outbreak of coronavirus disease 2019 (COVID-19) has resulted in more than 100 000 infected individuals in China and worldwide, there are few reports on the association of severe acute respiratory syndrome coronavirus 2 (SARS-CoV-2) with ocular abnormalities. Understanding ocular manifestations of patients with COVID-19 by ophthalmologists and others may facilitate the diagnosis and prevention of transmission of the disease.

**Objective:**

To investigate ocular manifestations and viral prevalence in the conjunctiva of patients with COVID-19.

**Design, Setting, and Participants:**

In this case series, patients with COVID-19 treated from February 9 to 15, 2020, at a hospital center in Hubei province, China, were retrospectively reviewed for ocular manifestations. During the period of treatment, the ocular signs and symptoms as well as results of blood tests and reverse transcriptase–polymerase chain reaction (RT-PCR) from nasopharyngeal and conjunctival swabs for SARS-CoV-2 were noted and analyzed.

**Main Outcomes and Measures:**

Ocular signs and symptoms as well as results of blood tests and RT-PCR for SARS-CoV-2.

**Results:**

Of the 38 included patients with clinically confirmed COVID-19, 25 (65.8%) were male, and the mean (SD) age was 65.8 (16.6) years. Among them, 28 patients (73.7%) had positive findings for COVID-19 on RT-PCR from nasopharyngeal swabs, and of these, 2 patients (5.2%) yielded positive findings for SARS-CoV-2 in their conjunctival as well as nasopharyngeal specimens. A total of 12 of 38 patients (31.6%; 95% CI, 17.5-48.7) had ocular manifestations consistent with conjunctivitis, including conjunctival hyperemia, chemosis, epiphora, or increased secretions. By univariate analysis, patients with ocular symptoms were more likely to have higher white blood cell and neutrophil counts and higher levels of procalcitonin, C-reactive protein, and lactate dehydrogenase than patients without ocular symptoms. In addition, 11 of 12 patients with ocular abnormalities (91.7%; 95% CI, 61.5-99.8) had positive results for SARS-CoV-2 on RT-PCR from nasopharyngeal swabs. Of these, 2 (16.7%) had positive results for SARS-CoV-2 on RT-PCR from both conjunctival and nasopharyngeal swabs.

**Conclusions and Relevance:**

In this study, one-third of patients with COVID-19 had ocular abnormalities, which frequently occurred in patients with more severe COVID-19. Although there is a low prevalence of SARS-CoV-2 in tears, it is possible to transmit via the eyes.

## Key Points

**Question:**

What are the ocular manifestations and conjunctival viral prevalence in patients from Hubei province, China, with coronavirus disease 2019 (COVID-19)?

**Findings:**

In this case series including 38 patients with COVID-19, 12 patients had ocular manifestations, such as epiphora, conjunctival congestion, or chemosis, and these commonly occurred in patients with more severe systemic manifestations. Reverse transcriptase–polymerase chain reaction results were positive for severe acute respiratory syndrome coronavirus 2 in 28 nasopharyngeal swabs and 2 conjunctival swabs, and more significant changes in blood test values appeared in patients with ocular abnormalities.

**Meaning:**

These data may assist ophthalmologists and others to understand the ocular manifestations of COVID-19, thus enhancing the diagnosis and prevention of the transmission of the disease.

## Teaser

This case series investigates the ocular manifestations and viral prevalence in the conjunctiva of patients with coronavirus disease 2019 (COVID-19).

## Introduction

Since December 2019, coronavirus disease 2019 (COVID-19) has been reported among patients in China. Currently, the disease is quickly spreading worldwide. The pathogen of COVID-19 is a novel coronavirus (severe acute respiratory syndrome coronavirus 2 [SARS-CoV-2]), identified as a member of the Coronaviridae family. Another coronavirus, named SARS-CoV-1, was responsible for severe acute respiratory syndrome.^1^ Compared with SARS-CoV-1, SARS-CoV-2 has a similar binding receptor and similar pathologic features systemically and epidemiological characteristics.^1,2^ Although there is no direct evidence that SARS-CoV-1 replication results in conjunctivitis and other ocular diseases, reports have emphasized the eye as a potential site for virus transmission.^3^ Similarly, SARS-CoV-2 transmission through the eye has been suspected.

Nevertheless, there are no reports in the medical literature at this time, to our knowledge, that identify a direct relationship between SARS-CoV-2 and the eye. Researchers have not reported ocular abnormalities nor have they stated in the medical literature if there was conjunctivitis or viral presence detected in the tears of patients with COVID-19. The objective of this study was to evaluate ocular involvement systematically in patients highly suspected of having or confirmed to have COVID-19.

## Methods

From February 9 to 15, 2020, patients with COVID-19 hospitalized in Yichang Central People’s Hospital were diagnosed based on the 5th edition of the National Guideline on Prevention and Control of the Novel Coronavirus Pneumonia (PC-NCP) published by the National Health Commission of China on February 8, 2020.^4^ The patient symptoms, ocular manifestations, chest computed tomographic scans, and results of blood tests and reverse transcriptase–polymerase chain reaction (RT-PCR) from nasopharyngeal and conjunctival swabs for SARS-CoV-2 were noted and analyzed. This study was approved by the ethics committee of Yichang Central People’s Hospital, and all patients gave written informed consent. All statistical analyses were performed using SPSS version 13.0 (SPSS Inc). Means for continuous variables were compared using independent-group *t* test when the data were normally distributed; otherwise, the Mann-Whitney test was used. Proportions for categorical variables were compared using the χ^2^ and Fisher exact test as appropriate. For unadjusted comparisons, a 2-sided α of less than .05 was considered statistically significant.

## Results

Of the 38 consecutive patients with COVID-19 who were recruited, 25 (65.8%) were male, and the mean (SD) age was 65.8 (16.6) years (Table 1). Among them, 28 patients (73.7%) had positive findings for COVID-19 on RT-PCR from nasopharyngeal swabs, and of these, 2 patients (5.2%) yielded positive findings for SARS-CoV-2 in their conjunctival as well as nasopharyngeal specimens. The other 10 patients who were hospitalized were judged to have COVID-19 by the guideline of PC-NCP,^4^ with fever and/or respiratory symptoms and lung computed tomography imaging features of COVID-19 pneumonia.

**Table 1.** Clinical Laboratory Results of Patients With Coronavirus Disease 2019 (COVID-19)

Measure	Mean (SD)			Difference (95% CI)	*P* value
	Total (N = 38)	Ocular symptoms			
		Yes (n = 12)	No (n = 26)		
Age, median (IQR), y	68 (53 to 76)	67 (52 to 76)	70 (62 to 79)	−3.39 (−8.47 to 15.25)	.28
Male, No. (%)	25 (65.8)	7 (58.3)	18 (69.2)	−0.11 (−0.44 to 0.22)	.51
Severe type, No. (%)^a^	15 (39.4)	8 (66.7)	7 (26.9)	0.40 (0.08 to 0.71)	.33
White blood cell count, /μL	7360 (4480)	10 900 (5580)	5730 (2690)	5160 (2460 to 7860)	.009
Lymphocyte count, /μL	890 (500)	710 (480)	980 (490)	−270 (−610 to 70)	.12
White blood cell count to lymphocyte count ratio	14.96 (20.23)	26.20 (25.36)	9.77 (15. 30)	16.43 (3.02 to 29.85)	.06
Neutrophil count, /μL	5920 (4640)	9510 (5820)	4260 (2820)	5250 (2430 to 8070)	.01
Monocyte count, /μL	500 (210)	620 (280)	440 (150)	170 (30 to 3100)	.06
Platelet count, ×10^3^/μL	184.39 (77.28)	184.58 (89.70)	184.31 (72.80)	0.28 (−55.18 to 55.73)	.99
PCT ≥0.05 ng/mL, No. (%)	15 (40.5)	8 (66.7)	7 (28.0)^b^	0.39 (0.06 to 0.71)	.03
CRP, mg/dL	5.17 (6.30)	8.55 (8.87)	3.61 (4.02)	4.95 (0.7 to 9.15)	.04
D-dimer, μg/mL	1.76 (2.42)	2.96 (3.93)^c^	1.35 (1.53)^d^	1.62 (−0.35 to 3.59)	.15
Creatine kinase, U/L	101.82 (85.81)	91.08 (58.35)	106.77 (96.53)	−15.69 (−77.03 to 45.66)	.61
Creatine kinase–MB, ng/mL	11.87 (5.67)	12.42 (4.94)	11.62 (6.05)	0.80 (−3.26 to 4.86)	.35
LDH, U/L	281.11 (154.47)	381.7 (196.52)	234.65 (105.89)	147.10 (48.04 to 246.15)	.03
Alanine aminotransferase, U/L	31.08 (27.46)	39.83 (45.25)	27.04 (12.69)	12.79 (−6.43 to 32.02)	.36
Aspartate aminotransferase, U/L	35.58 (26.58)	45.33 (41.73)	31.08 (14.58)	14.26 (−4.20 to 32.71)	.27
Urea nitrogen, mg/dL	41.24 (128.83)	20.21 (15.37)	50.94 (155.41)	−30.72 (−122.58 to 61.14)	.50
Creatinine, mg/dL	1.61 (2.84)	1.94 (3.81)	1.46 (2.34)	0.48 (−1.55 to 2.52)	.63

Abbreviations: CRP, C-reactive protein; IQR, interquartile range; LDH, lactate dehydrogenase; PCT, procalcitonin.

SI conversion factors: To convert white blood cell count to ×10^9^ per liter, multiply by 0.001; lymphocyte count to ×10^9^ per liter, multiply by 0.001; neutrophil count to ×10^9^ per liter, multiply by 0.001; monocyte count to ×10^9^ per liter, multiply by 0.001; platelet count to ×10^9^ per liter, multiply by 1; CRP to milligrams per liter, multiply by 10; D-dimer to nanomoles per liter, multiply by 5.476; creatine kinase to microkatals per liter, multiply by 0.0167; creatine kinase–MB to micrograms per liter, multiply by 1; LDH to microkatals per liter, multiply by 0.0167; alanine aminotransferase to microkatals per liter, multiply by 0.0167; aspartate aminotransferase to microkatals per liter, multiply by 0.0167; urea nitrogen to millimoles per liter, multiply by 0.357; and creatinine to micromoles per liter, multiply by 88.4.

^a^ Includes severe and critical cases of COVID-19.

^b^ Data from 1 patient missing.

^c^ Data from 4 patients missing.

^d^ Data from 3 patients missing.

A total of 12 of 38 patients (31.6%; 95% CI, 17.5-48.7) had ocular manifestations consistent with conjunctivitis, including conjunctival hyperemia, chemosis, epiphora, and increased secretions (Table 2). Among these 12 patients, there were 4 cases judged as moderate, 2 cases judged as severe, and 6 cases judged as critical, which was graded according to the guideline of PC-NCP^4^: moderate indicated fever and/or respiratory symptoms and lung computed tomography imaging findings; severe indicated dyspnea (respiratory frequency of 30 cycles per minute or greater), blood oxygen saturation of 93% or less, and an arterial partial pressure of oxygen to fraction of oxygen inspiration ratio of 300 or less; and critical indicated respiratory failure or shock or multiple organ dysfunction/failure.^4^ In these patients, 1 patient experienced epiphora as the first symptom of COVID-19. None of them experienced blurred vision. By univariate analysis, patients with ocular symptoms were more likely to have higher white blood cell and neutrophil counts and higher levels of procalcitonin, C-reactive protein, and lactate dehydrogenase than patients without ocular symptoms (Table 1). In addition, 11 of 12 patients with ocular abnormalities (91.7%; 95% CI, 61.5-99.8) had positive results for SARS-CoV-2 on RT-PCR from nasopharyngeal swabs. Of these, 2 (16.7%) had positive results for SARS-CoV-2 on RT-PCR from both conjunctival and nasopharyngeal swabs.

**Table 2.** Characteristics of 12 Patients With Ocular Manifestations

Patient No./Sex/Age, y	Temperature at ocular examination, °C	Respiratory symptoms	Clinical type^a^	Ocular manifestations	SARS-CoV-2 RNA test result	
					Nasopharyngeal swab	Conjunctival swab
1/F/80s	38.0	Dyspnea	Severe	Chemosis, epiphora	Positive	Negative
2/M/70s	38.0	Cough, expectorate	Critical	Secretion	Positive	Negative
3/M/50s	39.9	Cough, expectorate	Critical	Conjunctival hyperemia, secretion	Positive	Positive
4/F/80s	39.0	Dyspnea	Severe	Conjunctival hyperemia, chemosis, epiphora, secretion	Positive	Negative
5/F/60s	36.8	Cough	Critical	Chemosis, epiphora	Positive	Positive
6/M/60s	38.7	Cough, expectorate	Critical	Chemosis, epiphora, secretion	Positive	Negative
7/F/80s	36.5	None	Moderate	Chemosis, epiphora, secretion	Positive	Negative
8/F/70s	38.0	Cough	Critical	Chemosis, epiphora, secretion	Positive	Negative
9/M/60s	38.1	None	Critical	Chemosis, secretion	Positive	Negative
10/M/30s	39.6	Chest tightness	Moderate	Chemosis	Positive	Negative
11/M/40s	37.1	Cough	Moderate	Conjunctival hyperemia	Negative	Negative
12/M/70s	36.9	None	Moderate	Epiphora	Positive	Negative

Abbreviations: F, female; M, male; SARS-CoV-2, severe acute respiratory syndrome coronavirus 2.

^a^ Graded by the National Guideline on Prevention and Control of the Novel Coronavirus Pneumonia.^4^

## Discussion

Few previous investigations have evaluated ocular signs and symptoms in patients infected with SARS-CoV-1 and SARS-CoV-2. A few reports have evaluated for the presence of SARS-CoV-2 in tear fluid.^3,5^ Our investigation suggests that among patients with COVID-19, 31.6% (95% CI, 17.5-48.7) have ocular abnormalities, with most among patients with more severe systemic manifestations or abnormal findings on blood tests. These results suggest that ocular symptoms commonly appear in patients with severe pneumonia.

Our results show a low prevalence (5.2%; 95% CI, 0.6-17.8) of SARS-CoV-2 nucleotides in conjunctival specimens of patients with COVID-19, consistent with previous studies on severe acute respiratory syndrome.^3^ Of note, we found only 1 patient presenting with conjunctivitis as the first symptom. Previous reports have shown the shedding of potentially infectious virus can occur in people who have no fever and minor or absent signs of infection.^6^ Because unprotected eyes were associated with an increased risk of transmission of SARS-CoV-1,^7^ in support of our current results, our results might suggest that SARS-CoV-2 might be transmitted through the eye.

Limitations of this study include a relatively small sample size and absence of detailed ocular examinations to exclude intraocular disease owing to the logistical challenges of managing these patients at this time. In addition, we only sampled once from the eye of each patient, which can decrease the prevalence owing to false-negatives. Regardless, these preliminary results are shared in an effort to inform ophthalmologists and others around the world regarding ocular symptoms with COVID-19.
